# Intelligence

**Published:** 1826-07

**Authors:** 


### INTELLIGENCE.

### MONTHLY REPORT OF PREVALENT DISEASES.

During the latter part of last month (May) much rain fell, and the weather was comparatively cold; at this time numerous cases of rheumatism occurred, which, if not coming up to our idea of "rheumatic fever," were, nevertheless, in many instances sufficiently acute,—being attended with swelling, and occasionally with redness of the parts. On the sudden increase of temperature which took place at the beginning of June, a corresponding influence was produced upon the prevalent diseases, and it occurred to the writer to meet with many instances in quick succession, in which there was pain in the head, and giddiness, the last symptom being the most prominent and general. These constituted legitimate examples of determination to the head, and were speedily relieved by cupping on the back of the neck and free purging: the benefit derived from local bleeding was very decidedly superior to that resulting from a corresponding quantity abstracted by venesection. In only one instance within the notice of the writer did these symptoms terminate in apoplectic seizure. This occurred in the person of a gentleman of plethoric habit, and having the "make" which is regarded as predisposing to apoplexy. The fit was deep,—the pupils wide and dilated, and unaltered by bright light,—the breathing so feeble as to require the application of a mirror before the mouth to ascertain its existence;—no stertor,—the pulse about sixty, full; twenty ounces of blood were taken from the arm and sixteen from the back of the neck, after which the patient spoke and swallowed some purgative medicine. Another practitioner deemed it proper to take fifty ounces more blood from the arm within two hours—the entire quantity lost thus amounting to *eighty-six ounces.* The patient remained in a state of great exhaustion, and was rather incoherent, for several days, but ultimately recovered without any paralytic affection.

After the hot weather had continued for about eight or ten days a considerable number of cases of low febrile affection occurred; they were attended with much languor, foul tongue, loss of appetite, and sense of uneasiness, generally aggravated by pressure in the region of the liver. The treatment has consisted of from five to ten grains of calomel at night, followed by senna and salts next morning. Measles, Scarlatina, and Hooping-Cough have been met with, but scarcely in such numbers, with regard to any of them, as to constitute an epidemic.

*June* 23, 1826.

---

### *Lectures at the College of Physicians.*

Early in last May the theatre of the new College in Pall Mall East was opened for the purpose of resuming the annual lectures, of which the delivery has been necessarily suspended during the removal into the new edifice. The first were the Gulstonian lectures, which were delivered by Dr. Hawkins. The subject for the present year was Rheumatism, with the affections to which it gives rise of the heart and other internal organs. In the first lecture Dr. Hawkins gave a brief anatomical and pathological view of the different structures which appear to be the seat of rheumatism.

The second treated of the several forms and modifications of the disease, and much stress was laid upon the difference which may be observed in the local and constitutional symptoms of the disorder when situated in the fibrous textures, and when it attacks the synovial membranes.

The third lecture was devoted to the rheumatic affections of the heart and pericardium, the membranes of the brain, and structure of the eye.

These Lectures are advertised for publication.

Dr. Paris's lectures related to those chemical discoveries of the last twenty years, which have advanced our knowledge of the materia medica, and improved the practice of physic. They commenced on Friday the 12th of May, and were continued every succeeding Wednesday and Friday at the same hour.

Lecture I.—Friday, May 12th.—Introductory observations.—The theory of Lavoisier modified, but not subverted by modern discoveries.—The composite nomenclature of the French school requires reform.—*Discovery of Galvanism.*—Simple and compound galvanic circles.—Chemical powers of the voltaic pile.——Sir H. Davy's celebrated memoir on the chemical agencies of electricity—Electrical views of Berzelius and Oersted.

Lecture II.—Wednesday, May 17th.—The discoveries of Sir H. Davy, announced in his Bakerian Lecture, in 1808, form a new era in chemical science.—*Decomposition of the fixed alkalies*—Potassium—Sodium.—The *atomic doctrine,* or theory of definite proportionals—Discoveries to which it has given origin.—Dr. Wollaston's scale.—Practical applications of the doctrine of equivalents to pharmaceutic processes.

Lecture III.—Friday, May 19th.—Sir H. Davy's important views respecting the composition of muriatic acid.—*Chlorine.*—Its compounds with oxygen.—The Lecturer's opinions with regard to the composition of certain bodies rectified by the preceding discoveries.—*Iodine.*—Hydriodic acid.—Hydriodates.

Lecture IV.—Wednesday, May 24th.—The true nature of *prussic acid* ascertained by Guy-Lussac.—*Cyanogen.*—Preparation of *hydrocyanic acid,* according to the formula of Scheele and Vauquelin.—Its effects upon the animal system, and its value as a remedy.—The absorption of gaseous bodies by certain solid substances.—Condensation of the gases.—Cold produced by vaporization.—Oxygenated water of Thenard.

Lecture V.—Friday, May 26th.—On the extensive agency of water in chemical combination.—*Hydrates.*—New theory of the formation of lead plaster.—Discoveries connected with the composition and habitudes of *alcohol.*—*Ether.*—Recent experiments upon its nature and formation.—Various species of it produced by the action of different bodies on alcohol.

Lecture VI.—Wednesday, May 31st.—Recent discoveries in vegetable chemistry.—Extended series of proximate principles—Their conversion into each other.—*Analysis of opium.*—Morphia.—Meconic acid.—Narcotine.—Salts of morphia.—Analyses of the several species of *Peruvian bark.*—Cinchonia—Quina.—The habitudes of their respective salts.—Salifiable bases of other active vegetables.

Lecture VII.—Friday, June 2nd. — Veratria. — Atropria.—Hyoscyama.—Emeta—Lupulin—Elatin, &c.—Analyses of several medicinal vegetables.

Lecture VIII.—Wednesday, June 7th.—Experiments relative to the composition of the atmosphere.—*Eudiometry.*—Mr. Daniel's hygrometer.—The means of detecting the presence of animal effluvia—A new train of research proposed for that purpose.—*Miscellaneous notices.*

Specimens of the newly-discovered substances were exhibited, and the various subjects illustrated by numerous experiments.

On Monday, June 26th, the Harveian Oration was delivered by Dr. Warren.

All these lectures were numerously attended,—in some instances many were unable to gain admission from the crowded state of the theatre.

---

*Prize Question.*

The Academy of Medicine in Paris have proposed a prize for the following question :—"To appreciate, by positive observations, the more or less destructive action which the emanations resulting from the exercise of certain industrious professors cause on the constitution, and to discover the best remedies for them." The academy is aware that being announced in such general terms the proposed problem embraces an infinite variety of objects, and that it opens almost a boundless field for physical, chemical, and physiological researches, which it would be necessary to unite before arriving at the solution of the question. The academy does not expect to obtain at first a finished work on so extended and complicated a subject, it only wishes to excite the zeal of learned men of all classes; it expects from them some positive results on certain kinds of industrious employment, and to fix more clearly the ideas on that subject, it cites the following cases; viz. 1st. Those where mechanical causes act in a destructive manner on the organs of respiration, as particles floating in the atmosphere caused by the spinning of cotton or wool, or by the sawing of hard woods, &c. 2nd. Those where there is an absorption of very attenuated or dangerous matter, as mercury in some officinal preparations, lead or its oxides, or copper, or arsenic. 3d. Those where gases exercise on organs a destructive influence,—as chlorine on hydrochloric acid, so much used in the present day—the action of nitrous gas or nitric acid—or of ammonia, which is disengaged more or less pure in a great number of decompositions of animal matter; the action of sulphuretted hydrogen, which has destroyed so many scavangers. The prize is 1000 francs, and will be determined at the public meeting of 1828. Essays on the proposed subject must be sent to the office of the Academy, Rue de Poitiers, No. 8, before the 1st of February, 1828.

### METEOROLOGICAL JOURNAL,

*From May 20th, to June 20th,* 1826.

By Messrs. Harris and Co. Mathematical Instrument Makers, 50, High Holborn.

| May | Moon. | Rain gauge | Thermom. 9 A.M. | Thermom. MAX. | Thermom. MN. | Barometer. 9 A.M. | Barometer. 10P.M. | De Luc's Hygrom. 9 A.M. | De Luc's Hygrom. 10P.M. | Winds. 9 A.M. | Winds. 10P.M. | Atmospheric Variations. 9 a.m. | Atmospheric Variations. 2 p.m. | Atmospheric Variations. 10 p.m. |
|---|---|---|---|---|---|---|---|---|---|---|---|---|---|---|
| 20 | | | 56 | 63 | 47 | 29.63 | 29.85 | 73 | 63 | SE | NE | Cloudy | Fine | Fair |
| 21 | ○ | | 55 | 67 | 50 | 29.90 | 30.03 | 74 | 69 | N | N | Fine | — | Fine |
| 22 | | | 60 | 72 | 49 | 29.93 | 29.93 | 66 | 66 | NNE | ENE | — | — | — |
| 23 | | | 64 | 70 | 53 | 29.85 | 29.75 | 60 | 76 | NE | NNW | — | — | — |
| 24 | | .53 | 57 | 66 | 52 | 29.70 | 29.66 | 76 | 87 | NNW | NNE | Overca. | Rain | Rain |
| 25 | | | 54 | 59 | 53 | 29.57 | 29.52 | 87 | 90 | N | NNE | S. Rain | — | Fair |
| 26 | | | 58 | 62 | 54 | 29.55 | 29.66 | 82 | 80 | E | E | Fair | Fair | Rain |
| 27 | | .15 | 60 | 66 | 52 | 29.68 | 29.75 | 77 | 84 | ENE | ENE | Cloudy | Show'ry | Fair |
| 28 | ☾ | | 61 | 64 | 51 | 29.74 | 29.67 | 71 | 84 | NNE | N | — | — | Rain |
| 29 | | | 52 | 55 | 50 | 29.68 | 29.76 | 82 | 94 | N | NNE | Rain | Rain | — |
| 30 | | 1.35 | 53 | 55 | 51 | 29.75 | 29.80 | 94 | 95 | NW | NE | — | — | — |
| 31 | | | 54 | 59 | 51 | 29.80 | 29.75 | 88 | 89 | ENE | E | Overca. | Fair | Cloudy |
| June | | | | | | | | | | | | | | |
| 1 | | | 53 | 61 | 52 | 29.74 | 29.74 | 84 | 86 | NE | NNE | Rain | — | Rain |
| 2 | | .56 | 53 | 56 | 49 | 29.75 | 29.85 | 90 | 84 | NE | N | — | — | Fair |
| 3 | | | 63 | 65 | 52 | 29.92 | 30.05 | 67 | 63 | NW | NW | Fine | Fine | — |
| 4 | | | 64 | 68 | 54 | 30.06 | 30.08 | 64 | 76 | W | WNW | — | — | — |
| 5 | ● | | 63 | 67 | 55 | 30.14 | 30.18 | 66 | 65 | NNE | ESE | — | — | Fine |
| 6 | | | 59 | 71 | 59 | 30.16 | 30.08 | 68 | 69 | SE va. | NNW | — | — | Cloudy |
| 7 | | | 62 | 67 | 57 | 30.09 | 30.09 | 67 | 79 | NW | NNE | — | — | Fair |
| 8 | | | 63 | 69 | 55 | 30.10 | 30.05 | 66 | 64 | NE | NNE | — | — | — |
| 9 | | | 68 | 75 | 57 | 29.89 | 29.84 | 63 | 69 | NE | NNE | — | — | — |
| 10 | | | 62 | 74 | 56 | 29.83 | 29.90 | 67 | 76 | ENE | N | — | — | — |
| 11 | | | 63 | 74 | 60 | 29.94 | 30.02 | 73 | 75 | NNE | SE | — | — | Fine |
| 12 | | | 68 | 77 | 63 | 30.10 | 30.13 | 68 | 68 | NE | SE | — | — | — |
| 13 | ☽ | | 74 | 79 | 63 | 30.12 | 30.11 | 59 | 66 | NW | W | — | — | — |
| 14 | | | 69 | 80 | 63 | 30.11 | 30.08 | 67 | 66 | W | W | — | — | — |
| 15 | | | 72 | 78 | 55 | 30.02 | 29.95 | 60 | 62 | W | NNW | — | — | — |
| 16 | | | 64 | 68 | 55 | 30.15 | 30.12 | 55 | 60 | N | SSW | — | Cloudy | Cloudy |
| 17 | | | 66 | 73 | 62 | 30.24 | 30.15 | 60 | 64 | NW | WNW | — | Fine | Fine |
| 18 | | | 75 | 78 | 67 | 30.15 | 30.18 | 62 | 63 | NW | N | — | — | — |
| 19 | ○ | | 73 | 75 | 59 | 30.24 | 30.30 | 64 | 67 | E | SSE | Cloudy | Fair | — |

The quantity of Rain fallen in the month of May, was 2 inches 35.100ths.

